# Bioequivalence of a Donepezil/Memantine 10/20 mg Fixed‐Dose Combination Versus Single‐Component Tablets in Healthy Korean Males

**DOI:** 10.1002/cpdd.1556

**Published:** 2025-05-30

**Authors:** Minkyu Choi, Byung Hak Jin, Do Hoon Keum, Kyoung Hoon Mo, Min Soo Park, Juhwan Lee, Sungjin Park, Choon Ok Kim

**Affiliations:** ^1^ Department of Clinical Pharmacology Severance Hospital Yonsei University College of Medicine Seoul South Korea; ^2^ Department of Pharmaceutical Medicine and Regulatory Sciences Colleges of Medicine and Pharmacy Yonsei University Incheon South Korea; ^3^ Department of Pediatrics Yonsei University College of Medicine Seoul South Korea; ^4^ Department of Clinical Research Hyundai Pharmaceutical Co. Ltd. Seoul South Korea

**Keywords:** Alzheimer's disease, donepezil, fixed‐dose combination, memantine

## Abstract

A fixed‐dose combination (FDC) tablet formulation of donepezil/memantine (10/20 mg) was developed to improve medication compliance in patients with Alzheimer's disease (AD). This study compared the pharmacokinetic (PK) characteristics and safety profiles of an FDC formulation (donepezil/memantine [10/20 mg]) and single components (SCs) of donepezil (10 mg) and memantine (20 mg). In a randomized, open‐label, single‐dose, 2‐way crossover study, 24 healthy Korean participants received a single oral dose of FDC in 1 period and an SC of donepezil and memantine in another period. For PK analysis, blood samples were collected up to 240 hours after administration. The geometric mean ratios and their 90% confidence intervals for the main PK parameters (C_max_ and AUC_last_) indicated PK equivalence between the FDC and SC formulations. Regarding the safety profile, all adverse events were mild, with no serious adverse events. These findings support the use of an FDC formulation as a viable alternative to SCs of donepezil and memantine, potentially improving treatment adherence in patients with moderate‐to‐severe AD.

Alzheimer's disease (AD) is a progressive neurodegenerative disorder and a leading cause of dementia worldwide. It is characterized by cognitive impairment, behavioral disturbances, and functional decline.^1^, ^2^ With an increase in the aging global population, the prevalence of AD continues to increase steadily, posing significant challenges to healthcare systems and society.^3^ Pharmacological intervention remains crucial for AD management, particularly that targeting the cholinergic and glutamatergic systems, which play pivotal roles in cognition and neuroprotection.^4^, ^5^

Donepezil, a cholinesterase inhibitor, is widely prescribed for the management of mild‐to‐severe AD. Donepezil enhance cholinergic transmission by inhibiting the breakdown of acetylcholine, as is well documented.^6^ Memantine, an N‐methyl‐d‐aspartate (NMDA) receptor antagonist, provides neuroprotection by modulating glutamatergic excitotoxicity, which is a pathological hallmark of AD.^7^ Its clinical efficacy in improving cognitive and functional outcomes in patients with AD has been consistently demonstrated.^8^ Previous studies have shown that the combination of donepezil and memantine synergistically addresses cholinergic deficiencies and glutamatergic dysregulation, significantly improving cognitive performance and functional capabilities in patients with moderate‐to‐severe AD.^9^, ^10^, ^11^ Real‐world evidence indicates that approximately 9% of patients with AD are treated with a combination of donepezil and memantine, especially as this has been associated with improved 5‐year survival compared to monotherapy or no treatment.^12^ Patients with dementia often experience a significant reduction in medication adherence.^13^

This study aimed to compare the PK and safety profiles of the fixed‐dose combination (FDC) tablet of donepezil/memantine (10/20 mg) with those of single components (SCs) of donepezil (10 mg) and memantine (20 mg).

## Method

### Ethical Approval

This study was conducted in accordance with the Declaration of Helsinki and the Korean Good Clinical Practice. The study protocol was approved by the Institutional Review Board (IRB) of Severance Hospital (Seoul, South Korea; IRB number: 4‐2022‐0677) and the Ministry of Food and Drug Safety. The study was registered at ClinicalTrials.gov (identifier number: NCT05804279). Written informed consent was obtained from all participants before screening and enrollment in the study.

### Selection of Study Participants

Twenty‐four healthy Korean male volunteers aged 19‐55 years were eligible to participate in this study. All volunteers had a body weight of >55 kg and their body mass index ranged from 18.5 to 27.0 kg/m^2^ at the time of screening. The health of all participants was screened and assessed by the investigators based on medical history, physical examination, vital signs, 12‐lead electrocardiography (ECG), and laboratory tests including hematology, serum chemistry, serology tests, and urinalysis.

Participants who met any of the following criteria were excluded from the study: clinically significant hypersensitivity or a history of hypersensitivity to any of the investigational products (IPs) used in this study and other drugs, such as piperidine derivatives; a history of or currently having a clinically significant disease or gastrointestinal disorder; and a history of genetic disorders, such as galactose intolerance, Lapp lactase deficiency, or glucose‐galactose malabsorption.

### Study Design

This was a randomized, phase 1, open‐label, single‐dose, 2‐way crossover clinical trial. The 2 treatments were a single‐dose of an FDC tablet containing donepezil 10 mg and memantine 20 mg (Hyundai Pharmaceutical Co., Ltd, Seoul, South Korea), referred to as FDC treatment, and co‐administration of single components of donepezil 10 mg and memantine 20 mg, referred to as SC treatment. The participants were randomly assigned to 1 of the 2 treatment sequence groups in a 1:1 ratio (Group 1, SC‐FDC treatment; Group 2, FDC‐SC treatment). A 21‐day washout period was implemented between the treatment periods. The 21‐day washout period was determined considering the terminal elimination half‐lives of donepezil and memantine, which are approximately 81.5 and 70.9 hours, respectively, and was intended to minimize the risk of carryover effects.^14^, ^15^ All participants received IPs with 150 mL of water after at least 10 hours of fasting. After administering IPs in each period, all participants were required to fast for at least 4 hours. In addition, all drugs, including over‐the‐counter medications and beverages that could affect the PKs of each IP, were prohibited during the study period.

### Plasma Drug Concentration Analysis

Serial blood samples were collected for PK evaluation of donepezil and memantine. During each treatment period, blood samples were obtained at pre‐dosing and 0.5, 1, 1.5, 2, 3, 4, 5, 6, 8, 10, 24, 48, 72, 144, 192, and 240 hours after the administration of each treatment. Approximately 10 mL of blood was drawn into an ethylenediaminetetraacetic EDTA‐K2‐coated blood‐collection tube to quantify the plasma concentrations of donepezil and memantine. The samples were centrifuged at 1800 g at 4°C for 15 minutes, within 60 minutes of collection. Plasma aliquots were stored at −70°C or lower until analysis. The plasma concentrations of donepezil and memantine were determined using a validated liquid chromatography‐tandem mass spectrometry (LC‐MS/MS) method.

For donepezil, plasma samples (100 µL) were transferred to a tube containing 10 µL of the internal standard (donepezil‐d₇, 100 ng/mL in 50% acetonitrile) and 300 µL of acetonitrile. After vortexing at 194.9 g for 5 minutes and centrifugation at 13,040 g for 5 minutes, 100 µL of the supernatant was transferred to a new microtube and diluted with 100 µL of water, followed by vortexing at 194.9 g for 30 seconds. A 2 µL aliquot of the supernatant was injected into the LC‐MS/MS system. Chromatographic separation was performed on a Unison UK‐C18 column (75 × 2.0 mm internal diameter, 3 µm) maintained at 40°C. The mobile phase consisted of 0.1% formic acid in 5 mM ammonium formate (A) and 0.1% formic acid in acetonitrile (B), delivered at a flow rate of 0.2 mL/min using the following gradient: 0.0‐1.5 minutes, 55:45 (A:B); 2.0‐3.5 minutes, 5:95; 3.6‐5.5 minutes, 55:45. The autosampler temperature was maintained at 15°C. LC‐MS/MS analysis was conducted using a Triple Quad 5500+ system (SCIEX, Framingham, MA, USA) equipped with electrospray ionization (ESI) in positive ion mode. Mass spectrometric detection was performed in multiple reaction monitoring (MRM) mode with the transitions of m/z 380.1 → 91.1 for donepezil and m/z 387.1 → 98.1 for donepezil‐d₇. Quantification was performed using the peak area ratio of the analyte to the internal standard. The calibration curve was linear over the concentration range of 0.1‐100 ng/mL with a 1/x^2^ weighting and the lower limit of quantification (LLOQ) was 0.1 ng/mL. Quality control (QC) samples were prepared at concentrations of 0.3 ng/mL (low), 20 ng/mL (medium), and 80 ng/mL (high). Intra‐batch accuracy ranged from 95.00% to 108.92% with coefficients of variation (CVs) from 0.12% to 2.05%, and inter‐batch accuracy ranged from 98.13% to 102.52% with CVs from 1.09% to 4.92%.

For memantine, plasma samples (100 µL) were transferred to a tube containing 10 µL of the internal standard (memantine‐d₆, 200 ng/mL in 50% acetonitrile) and 500 µL of 0.1% formic acid in methanol. After vortexing at 194.9 g for 1 minute and centrifugation at 13,040 g for 5 minutes, 2 µL of the supernatant was injected into the LC‐MS/MS system. Chromatographic separation was performed on a Poroshell 120 EC‐C18 column (50 × 3.0 mm internal diameter, 2.7 µm) maintained at 40°C. The mobile phase consisted of 0.1% formic acid in water (A) and methanol (B), mixed in a ratio of 45:55 (v/v) and delivered at a flow rate of 0.35 mL/min. The autosampler was maintained at 5°C and the total run time was 3 minutes. LC‐MS/MS analysis was carried out using a Triple Quad 6500 system (SCIEX, Framingham, MA, USA) with ESI in positive ion mode. MRM transitions were monitored at m/z 180.2 → 163.2 for memantine and m/z 186.2 → 169.2 for memantine‐d₆. Quantification was based on the peak area ratio of memantine to its internal standard. The calibration curve was linear over the range of 0.1‐100 ng/mL using a 1/x^2^ weighting and the LLOQ was 0.1 ng/mL. Quality control samples were prepared at 0.3 ng/mL (low), 25 ng/mL (medium), and 80 ng/mL (high). Intra‐batch accuracy ranged from 97.48% to 103.73% with CVs between 0.78% and 1.66%, and inter‐batch accuracy ranged from 99.62% to 103.17% with CVs from 1.28% to 2.90%. Data acquisition and processing for both analytes were performed using Analyst software version 1.7.1 (SCIEX, Framingham, MA, USA).

### Safety Assessment

All adverse events (AEs) were monitored during the study period. Safety assessments included physical examination, vital signs, 12‐lead ECGs, and laboratory tests, including hematology, serum chemistry, and urinalysis. Laboratory tests for AE assessment were conducted at discharge and during the follow‐up visit. All AEs were documented in accordance with MedDRA (version 25.1).

### Pharmacokinetic Analysis

PK analysis was conducted using data from the participants who completed the study. PK parameters were derived using a noncompartmental analysis method implemented with WinNonlin version 8.3 (Certara, Princeton, NJ, USA). Statistical computations were performed using SAS software version 9.4 (SAS Institute Inc., Cary, NC, USA).

The primary PK endpoints were the maximum concentration of the drug in the plasma (C_max_) and the area under the plasma drug concentration‐time curve to the last concentration (AUC_last_) for donepezil and memantine. The secondary endpoints were the AUC from time 0 to infinity (AUC_inf_), time to maximum plasma concentration (T_max_), elimination half‐life (t_1/2_), and apparent clearance (CL/F) of both compounds.

The actual blood sampling time was used for PK evaluation; missing values of individual plasma concentrations were not imputed. C_max_ was obtained from the measured data, whereas AUC was calculated using the trapezoidal method. PK comparisons of donepezil and memantine between the treatment groups were based on the geometric least squares mean ratios (GMRs) and 90% confidence intervals (90% CIs) for C_max_ and AUC_last_.

## Results

### Study Participants

A total of 24 healthy male participants were enrolled in this study, with 12 randomly assigned to each sequence group. Male subjects were exclusively enrolled to minimize inter‐individual PK variability, since hormonal fluctuations and sex‐specific physiological differences in female may influence the PK parameters. Four participants from Group 1 and 1 from Group 2 withdrew from the study. These participants were excluded from the PK analysis set but were included in the demographics and safety analysis. The demographic characteristics of the participants are presented in Table S1. There were no significant differences in the demographics between the 2 groups.

### Pharmacokinetics

**Figure 1:**
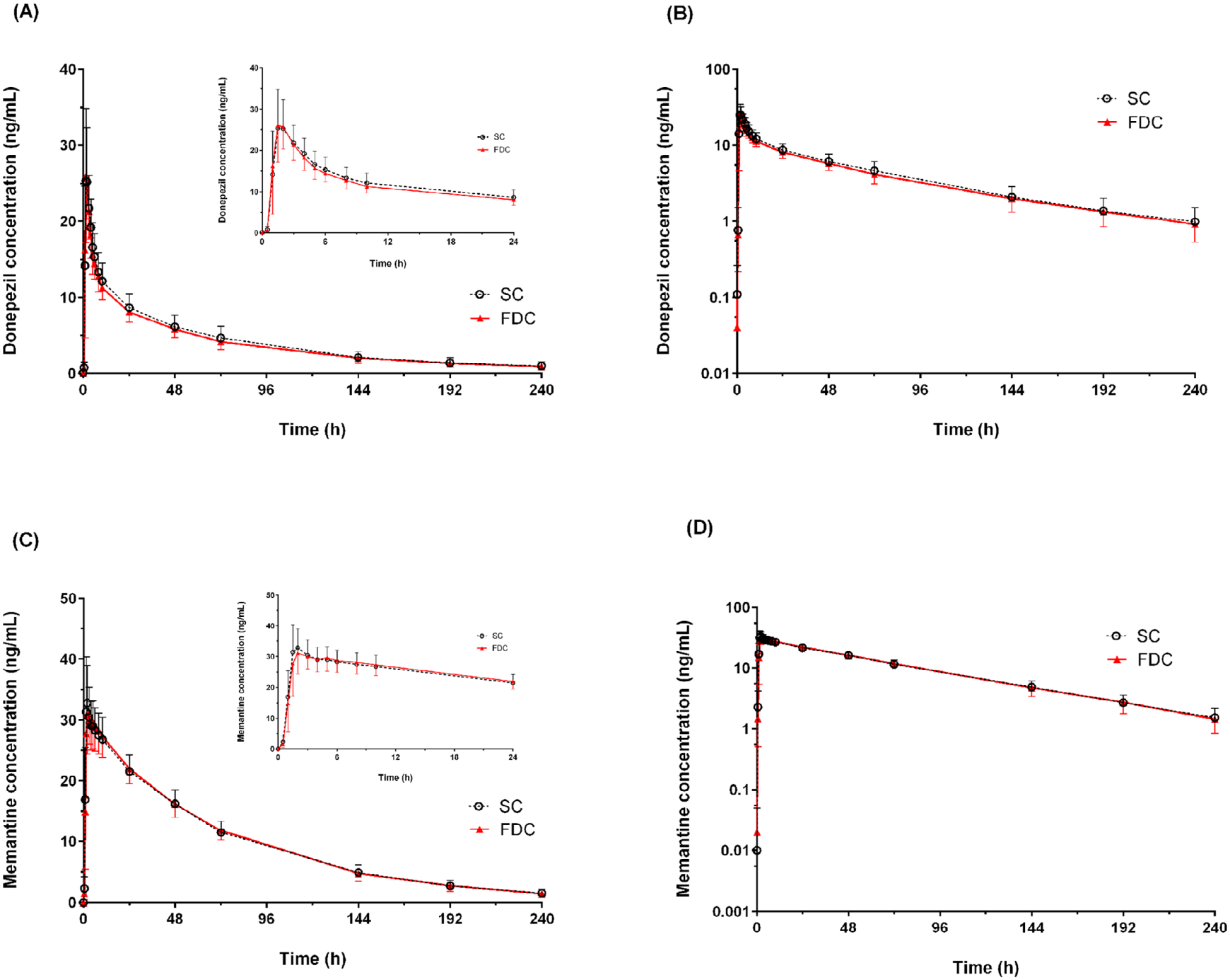
Mean (SD) plasma concentration‐time profiles of donepezil (A and B) and memantine (C and D) after single oral administration of a fixed‐dose combination tablet of donepezil/memantine 10/20 mg (FDC) or co‐administration of single components of donepezil 10 mg and memantine 20 mg (SC). (A, C) Linear scale; (B, D) semi‐logarithmic scale. Error bars represent standard deviation. FDC, fixed‐dose combination; SC, single components.

PK analysis was conducted on 19 participants who completed the study. The PK profiles following the administration of FDC and SC were comparable. The mean plasma concentration‐time profiles of donepezil and memantine for both treatments are shown in Figure 1 and the corresponding PK parameters are summarized in Table 1. The point estimate (90% CI) for the GMRs (FDC/SC) of C_max_ and AUC_last_ are presented in Table 2.

**Table 1:** Summary of the PK Parameters of Donepezil and Memantine

Donepezil	SC (n = 19)	FDC (n = 19)
C_max_ (ng/mL)	29.5 ± 6.0	29.7 ± 6.4
AUC_last_ (h•ng/mL)	976 ± 249	916 ± 194
AUC_inf_ (h•ng/mL)	1096 ± 333	1023 ± 242
T_max_ (hour)	1.5 (1.0, 4.0)	1.5 (1.0, 3.0)
t_1/2_ (hour)	76.1 ± 17.9	77.6 ± 12.5
CL/F (L/h)	9.9 ± 2.8	10.3 ± 2.4

Memantine	SC (n = 19)	FDC (n = 19)
C_max_ (ng/mL)	34.9 ± 6.2	34.1 ± 5.7
AUC_last_ (h•ng/mL)	2214 ± 312	2225 ± 303
AUC_inf_ (h•ng/mL)	2356 ± 355	2350 ± 345
T_max_ (hour)	2.0 (1.5, 8.0)	2.0 (1.5, 6.0)
t_1/2_ (hour)	57.8 ± 14.9	54.9 ± 12.0
CL/F (L/h)	8.7 ± 1.8	8.7 ± 1.7

AUC_last_, area under the plasma drug concentration‐time curve to last concentration; AUC_inf_, AUC from time 0 to infinity; CL/F, apparent clearance; C_max_, maximum concentration of the drug in plasma; FDC, fixed‐dose combination; SC, single components; T_max_, time to C_max_; t_1/2_, elimination half‐life.

Data are shown as mean ± standard deviation, except for T_max_, for which data are shown as median (minimum, maximum). FDC, a single‐dose administration of a fixed‐dose combination tablet of donepezil/memantine (10/20 mg); SC, a single‐dose of concomitant administration of donepezil (10 mg) and memantine (20 mg) as single components.

**Table 2:** Comparison of PK Parameters of Donepezil and Memantine

	GeoLSM			90% CI for GMR	
Parameter	SC	FDC	GMR	Lower limit	Upper limit
Donepezil					
C_max_ (ng/mL)	28.4	28.5	1.00	0.94	1.07
AUC_last_ (h•ng/mL)	926	885	0.96	0.93	0.99
Memantine					
C_max_ (ng/mL)	33.9	33.3	0.98	0.94	1.03
AUC_last_ (h•ng/mL)	2191	2191	1.00	0.97	1.03

AUC_last_, area under the plasma drug concentration‐time curve to the last concentration; CI, confidence interval; C_max_, maximum concentration of drug in plasma; FDC, fixed‐dose combination; GeoLSM, geometric least squares mean; GMR, geometric least squares mean ratio; SC, single components.

FDC, a single‐dose administration of a fixed‐dose combination tablet of donepezil/memantine (10/20 mg); SC, a single‐dose of concomitant administration of donepezil (10 mg) and memantine (20 mg) as single components.

### Safety and Tolerability

This study reported 44 AEs in 16 participants (Table S2). All AEs were defined as drug reactions. All AEs were mild and most patients recovered without complications. The AEs between the 2 treatment groups were similar and consistent with the known AE profiles of donepezil and memantine. The most common AEs were dizziness and nausea, with comparable incidence rates between the treatment groups.

## Discussion

This study compared the PK and safety profiles of FDC treatment (administration of FDC tablet of donepezil/memantine [10/20 mg]) and SC treatment (concomitant administration of separate tablets of donepezil [10 mg] and memantine [20 mg]). The PK results of this study demonstrated that the 90% CIs of the GMRs of C_max_ and AUC_last_ for donepezil and memantine fell within the bioequivalence criteria range of 0.80‐1.25.

In AD, memantine is commonly introduced as an add‐on therapy in the moderate stages after initiating acetylcholinesterase inhibitors in the earlier stages.^16^, ^17^ Clinical trials investigating the co‐administration of donepezil and memantine in patients with moderate‐to‐severe AD revealed superior outcomes in cognitive function, activities of daily living, overall clinical response, behavioral symptoms, and caregiver dependency compared to donepezil monotherapy.^10^, ^11^, ^18^ In clinical practice, the initiation and titration of these therapies are typically guided by disease severity. Donepezil is generally started at 5 mg once daily and increased to 10 mg after 4‐6 weeks, while memantine is initiated at 5 mg daily and titrated weekly by 5 mg to a target dose of 20 mg per day for the treatment of moderate‐to‐severe AD. At the maintenance phase, most patients with moderate‐to‐severe AD are maintained on donepezil 10 mg and memantine 20 mg,^19^ therefore an FDC of donepezil and memantine (10/20 mg) offers a practical benefit for AD patients who have already reached stable, target therapeutic doses of both single components. By consolidating 2 medications into a single tablet, FDCs reduce pill burden, simplify complex regimens, and are particularly valuable for patients with cognitive impairment, where adherence to multiple medications can be challenging. This streamlined approach may enhance long‐term treatment continuity and overall therapeutic outcomes. Research on other medical conditions requiring polypharmacy, such as diabetes and cardiovascular disease, has shown that transitioning to FDC can significantly improve medication adherence.^20^, ^21^ Since donepezil 10 mg and memantine 20 mg are commonly used as maintenance doses in patients with moderate‐to‐severe AD, an FDC containing these doses, which has demonstrated PK bioequivalence to the co‐administration of individual agents, may offer a valuable alternative formulation to improve treatment adherence in clinical practice.

The compatibility between donepezil and memantine is well‐established from a PK perspective. According to in vitro studies, memantine does not affect the efficacy of acetylcholinesterase inhibitors such as tacrine, galantamine, or donepezil.^22^ A previous study demonstrated that co‐administration of donepezil and memantine did not result in significant interactions between the 2 drugs compared to memantine monotherapy.^23^

Safety assessments of the FDC treatment demonstrated that the AE profile was closely aligned with that of the SC treatment. Both treatments were well‐tolerated without serious AEs. All AEs were mild and most patients recovered without complications. Among the reported AEs, nausea and dizziness were the most common, with comparable rates between the treatment groups.

This study indicated that the FDC of donepezil and memantine (10/20 mg) was generally well‐tolerated in healthy participants. However, similar to other phase 1 studies on anti‐dementia medications, this study involved healthy participants who, on average, were approximately 40 years younger than typical patients with AD, who often have multiple comorbidities, therefore the PK results of this study may not fully represent the AD patient population. Accordingly, a long‐term study is necessary to compare the pharmacodynamic effects and safety of FDC and SC treatments in patients with AD.

## Conclusion

This study demonstrated that the PK and safety profiles of FDC tablets of donepezil/memantine (10/20 mg) are comparable to those observed with the SCs of donepezil (10 mg) and memantine (20 mg).

## Conflicts of Interest

Juhwan Lee and Sungjin Park are employees of Hyundai Pharmaceutical Co. Ltd. The remaining authors declare that they have no conflicts of interest. The authors declare that the conflicts of interest declared by Juhwan Lee and Sungjin Park did not have any significant influence on the analysis and interpretation of the results in this study.

## Funding

This study was sponsored by Hyundai Pharmaceutical Co. Ltd., Seoul, Republic of Korea. The sponsor had no role in the design of the study, data collection and analysis, decision to publish, or preparation of the manuscript.

## Supporting information



Supporting Information

## Data Availability

The data supporting the findings of this study are available from the corresponding author upon reasonable request. However, certain data may be restricted due to privacy or ethical considerations.
