# Supplementary material for: Bioequivalence of a Donepezil/Memantine 10/20 mg Fixed‐Dose Combination Versus Single‐Component Tablets in Healthy Korean Males
Source: Clin Pharmacol Drug Dev. 2025 May 30;14(9):710–6. doi: 10.1002/cpdd.1556 (PMC12402880; doi:10.1002/cpdd.1556)
Supplement: Supplementary file 1 — Supporting Information [file CPDD-14-710-s001.docx]

**Supplementary Digital Contents**

Table S1. Demographic characteristics of study participants

| Variables^a^ | Group 1 (SC → FDC)  (n = 12) | Group 2 (FDC → SC)  (n = 12) | Total  (n = 24) | *p*-value^b^ |
| --- | --- | --- | --- | --- |
| Age (year) | 26.5 ± 6.9 | 26.2 ± 8.6 | 26.3 ± 7.6 | 0.92 |
| Weight (kg) | 71.6 ± 7.6 | 69.0 ± 9.5 | 70.3 ± 8.5 | 0.47 |
| Height (cm) | 174.6 ± 4.9 | 171.4 ± 6.7 | 173.0 ± 6.0 | 0.20 |
| BMI (kg/m^2^) | 23.5 ± 2.5 | 23.4 ± 2.0 | 23.4 ± 2.2 | 0.91 |

**Notes:** ^a^Data are shown as mean ± standard deviation; ^b^*p* -values were calculated using an independent *t-test* to compare differences between groups 1 and 2; FDC, a single-dose FDC tablet containing donepezil/memantine (10/20 mg); SC, a single-dose concomitant administration of donepezil (10 mg) and memantine (20 mg) as single components.

**Abbreviations:** BMI, body mass index; FDC, fixed-dose combination; SC, single components.

Table S2. Summary of adverse events (AE)

| System Organ Class/Preferred Term | Number of Participants (Number of AEs) | | |
| --- | --- | --- | --- |
|  | SC (n = 23) | FDC (n = 22) | Total (n = 24) |
| **Total** | **13 (23)** | **13 (21)** | **16 (44)** |
| **Gastrointestinal disorders** | **6 (7)** | **9 (9)** | **12 (16)** |
| Abdominal pain upper | 1 (1) | - |  |
| Nausea | 6 (6) | 9 (9) |  |
| **General disorders and administration site conditions** | **1 (1)** | **-** | **1 (1)** |
| Pyrexia | 1 (1) | - |  |
| **Infections and infestations** | **-** | **1 (1)** | **1 (1)** |
| Nasopharyngitis | **-** | 1 (1) |  |
| **Investigations** | **2 (3)** | **1 (2)** | **2 (5)** |
| Alanine aminotransferase increased | 1 (1) | 1 (1) |  |
| Aspartate aminotransferase increased | 1 (1) | - |  |
| Blood creatine phosphokinase increased | 1 (1) | - |  |
| Blood triglycerides increased | - | 1 (1) |  |
| **Nervous system disorders** | **10 (10)** | **9 (9)** | **12 (19)** |
| Dizziness | 10 (10) | 8 (8) |  |
| Headache | - | 1 (1) |  |
| **Renal and urinary disorders** | **2 (2)** | **-** | **2 (2)** |
| Hematuria | 1 (1) | **-** |  |
| Proteinuria | 1 (1) | **-** |  |

**Notes:** Data are shown as the number of participants who reported AE (the number of AE); FDC, a single-dose administration of a fixed-dose combination tablet of donepezil/memantine (10/20 mg); and SC, a single-dose of concomitant administration of donepezil (10 mg) and memantine (20 mg) as single components. **Abbreviations:** FDC, fixed-dose combination; SC, single components
